# Editorial for the Special Issue Entitled “Recent Advances in Crosslinked Gels”

**DOI:** 10.3390/gels10050310

**Published:** 2024-05-02

**Authors:** Melike Firlak Demirkan, John G. Hardy

**Affiliations:** 1 Department of Chemistry, Faculty of Science, Gebze Technical University, Gebze 41400, Turkey; 2 Department of Chemistry, Faraday Building, Lancaster University, Lancaster LA1 4YB, UK; 3 Materials Science Lancaster, Lancaster University, Lancaster LA1 4YW, UK

## 1. Introduction

A gel can be defined as a semi-solid structure that has mechanical properties ranging from tough to soft, depending on their constituents. When they are in a steady state, they show no flow; however, the liquid phase may diffuse in or out through the system, potentially absorbing solvent up to hundreds-fold of their mass. Polymeric chains physically or chemically crosslink to form crosslinked gels, and swellable polymeric networks are generated [1]. The polymer used for the formation of a crosslinked gel can be either natural (e.g., polysaccharides [2,3], proteins [4,5]) or synthetic (e.g., acrylates [6]), and in some cases, combinations of both can be used. The properties of the gels can be tuned to meet the needs of their intended applications (e.g., altering the polymer structures, gel composition, and/or stimulus responsivity) [7].

The applications of gels include the adsorption of pollutants for environmental remediation, drug delivery, sensors, tissue engineering, regenerative medicine, and more, and this Special Issue, entitled “Recent Advances in Crosslinked Gels”, in *Gels*, brings together valuable articles and reviews of expert researchers in the field from around the world. This Special Issue comprises nine articles and three reviews on the synthesis, crosslinking behavior, mechanical and rheological properties, and applications of crosslinked gels. 

## 2. Physical Properties of Gels

Chen and Huang investigated the crosslinking behavior of polyethylene resins. The researchers used a combination of computational model simulations and rheological studies, establishing the mathematical equation of an “S” model for predicting the crosslinking degree of PE resins. Crosslinking is crucial for the formation of three-dimensional gels, as is the degree of the crosslinking, which results in large property changes. Both physically and chemically crosslinked interpenetrated networks (IPN) were prepared by Dueramae and coworkers, and the impacts of the crosslinking degree on the mechanical properties of the gels were successfully demonstrated. Gelation time is an important descriptor of gels’ different characteristics (specifically the time required for a solution to reach its gel point), which is dependent on the thermodynamic conditions of the crosslinking process (i.e., temperature, concentration, functionality of the molecules, and multiplicity of the crosslink junctions), and Tanaka investigated the relationship between the gelation time and the parameters on which the crosslinking process depends.

Onoda-Yamamuro and coworkers investigated the mechanism of ice crystallization in Sephadex^®^ gels. Joly et al. reported semi-IPNs composed of crosslinked alginate/chitosan gels; their physicochemical properties were investigated in detail by using rheology, IR spectroscopy, and electron paramagnetic resonance (EPR) spectroscopy techniques. The results showed that the alginate network determined the entire system’s characteristics. All the techniques used for investigation of the physicochemical properties of the semi-IPN gels showed that the gels had different properties according to the presented crosslinker and the order in which they were added. 

## 3. Applications of Hydrogels

Baines et al. reported the application of whey protein isolate (WPI) hydrogels enriched with poly-ɣ-glutamic acid (γ-PGA) as a potential scaffold for bone tissue regeneration. Preosteoblastic cells were used for cytotoxicity assessments; the WPI-γ-PGA hydrogels were not found to be toxic, and they supported cell proliferation. Additionally, the authors reported that cell viability was significantly increased on WPI-γ-PGA scaffolds on day 14, compared to the WPI control scaffolds. 

Latif et. al. reported the application of nanoemulsion gel formulations for methotrexate release, with a view to treating skin infections.

Cheng and co-authors designed a new triblock copolymer-based hydrogel for sustained administration of the peptide drug calcitonin in vitro. 

The oral administration route is one of the most commonly used administration routes due to its convenience, ease of administration, and non-invasiveness. However, this route has some disadvantages such as low adsorption and bioavailability, and Hathout demonstrated via a meta-analysis study that polymeric nanoparticles play a critical role in enhancing the bioavailability of orally administered drugs, compared to the other conventional formulations. 

## 4. Reviews

This Special Issue includes three review articles that discuss the effects of crosslinking agents and applications of crosslinked gels on drug delivery and tissue engineering. Liu et al. compared the differences between conventional crosslinking agents and cyclodextrin-based polyrotaxanes. They report that the hydrogels prepared with conventional crosslinkers had fixed crosslinking points, while hydrogels prepared with polyrotaxane had freely sliding crosslinking points along the molecule, which provided gel flexibility, higher swellability, and better mechanical properties. Ghassemi et al. reviewed recent applications of extracellular vesicle (EV) technology for myocardial regeneration and discussed the use of hydrogels to improve the bioavailiability of EVs. Mashabela et al. reviewed hydrogel-based drug delivery devices to treat central nervous system (CNS) diseases, concluding that the combination of nanotechnology with crosslinked gels will improve the strategies for CNS disease treatments.

In conclusion, several interesting research and review articles on the recent advances in crosslinked hydrogels are collected together in this Special Issue, and we hope this issue will support the development of next-generation crosslinked gels with important applications.
